# The Influence of Meat Batter Composition and Sausage Diameter on Microbiota and Sensory Traits of Artisanal Wild Boar Meat Sausages

**DOI:** 10.17113/ftb.57.03.19.6197

**Published:** 2019-09

**Authors:** Ivica Kos, Ana Zgomba Maksimović, Marija Zunabović-Pichler, Sigrid Mayrhofer, Konrad J. Domig, Mirna Mrkonjić Fuka

**Affiliations:** 1 Department of Animal Science and Technology, University of Zagreb Faculty of Agriculture, Svetošimunska 25, 10000 Zagreb, Croatia; 2 Department of Microbiology, University of Zagreb Faculty of Agriculture, Svetošimunska 25, 10000 Zagreb, Croatia; 3 Department of Food Science and Technology, BOKU University of Natural Resources and Life Sciences Vienna, Muthgasse 18, 1190 Vienna, Austria

**Keywords:** wild boar sausages, sausage diameter, sensory evaluation, lactic acid bacteria, *Leuconostoc mesenteroides*, starter culture selection

## Abstract

In this study, the influence of meat batter composition and sausage diameter on the development of microbiota and sensory traits of traditional, spontaneously fermented wild boar meat sausages are evaluated. This research also demonstrates how principal component analysis (PCA) can be used to relate product sensory properties to particular microbial genotype and to select potential starter or adjunct culture. Generally, similar microbiological results were obtained in all types of products. The undesirable microbiota was either not detected at any sausage production stage or its number decreased below the detection limit in ripened sausages. The low growth rate of lactic acid bacteria (LAB) was consistent with the obtained pH and slow acidification rate. Although no differences in the composition of LAB species were noticed between sausage types (50S=50% wild boar meat in small casing, 50L=50% wild boar meat in large casing, 100S=100% wild boar meat in small casing), a clear separation based on LAB genotypes could be observed. Upon quantitative descriptive analysis, significant differences in sensory attributes between sausage types were established. According to the PCA, the overall acceptability traits of sausages are closely linked to one *Leuconostoc mesenteroides* genotype (LM_4). Of all tested technological properties, LM_4 strains showed remarkable acidification ability, lowering the pH from pH=5.41 to 3.74, and pronounced proteolytic activity on skimmed milk as well as antagonistic activity against *Staphylococcus aureus* (DSM 20231) and *Brochothrix thermosphacta* (LMG 17208). Lipolytic and haemolytic activities were not detected, and all analyzed strains were susceptible to tested antibiotics and possessed no biogenic amine genes.

## INTRODUCTION

Fermentation is one of the oldest technologies used to store food for a longer time period and it is an area where microbes, meat and technology meet. Artisanal wild boar sausages are manufactured mostly on the small-scale or household level, usually from a mixture of wild boar (*Sus scrofa* L.) meat and meat and back fat of domestic pig (*Sus scrofa domesticus* L.), in ratios varying according to the manufacturer’s recipe. Sausages are obtained following traditional recipes without the addition of starter cultures, thus completely relying on the fermentation capacity of the naturally present microbiota. Among this microbiota, lactic acid bacteria (LAB) have a central role in the fermentation by lowering the pH, and therefore inhibiting the proliferation of undesirable microorganisms as well as by providing a suitable environment for biochemical processes (*1*). Although certain species of LAB are associated with fermentation, the metabolic activity contributing to the development of final sensory characteristics is presumed to be strain (genotype) specific (*2*).

In addition to the meat used for sausage production and the naturally occurring microbiota in meat batter, previous studies have shown that the sausage diameter (size) can influence the sensory traits through the accumulation of volatile compounds (*3*). Furthermore, it was proposed that the influence of sausage diameter on the sensory traits is due to water loss kinetics, oxygen availability (*3*) and the duration of ripening period (*4*). These factors can also influence the growth and predominance of certain LAB strains during fermentation and ripening.

Physicochemical, microbiological and sensory traits of traditional spontaneously fermented sausages have been reported by several authors (*4*-*7*). However, published data for spontaneously fermented wild boar sausages are very limited (*8*). In particular, microbial data at genotype (strain) level and in relation to sensory traits are missing. Therefore, the main objective of this study is to determine the differences in microbiota and sensory traits of traditional dry wild boar meat sausages of different diameter and meat batter composition. Special focus is put on a detailed microbiological characterization of the predominant LAB and their relationship with sensory traits in order to select potential starter or adjunct cultures.

## MATERIALS AND METHODS

### Sausage preparation and pH measurement

**Fig. 1:**
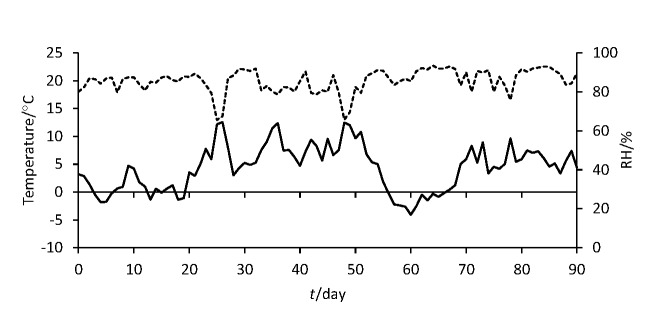
Temperature (solid line) and relative humidity (RH, dashed line) during sausage production

Three types of sausages were produced at the same small-scale production unit ’Ban Josip Jelačić‘ in Prolom, Croatia, each in triplicate, from three independent batches. Sausages were manufactured without the application of starter cultures and nitrites, as a mixture of wild boar and domestic pig meat in a 1:1 ratio (sausages 50S and 50L) or completely from wild boar meat (100S). Meat of 1.5 year old female animals was used, mainly because of the boar taint of uncastrated male wild boar, and more intense meat colour properties. The lean meat and fat ratio was approx. 80:20 in all sausage types. Lean meat was minced to a particle size of 8 mm and fat to 6 mm. The mixture was flavoured with salt (2.2%), white wine (1%), fresh garlic (0.3%), ground red chili peppers (0.3%), table sugar (0.3%) and ground black pepper (0.2%). The sausage mixture was filled in artificial collagen casings with a diameter of 35 mm (50S and 100S) or 50 mm (50L). The sausages were further manufactured in a drying chamber under varying environmental conditions with relative humidity from 65 to 93% (higher in the beginning) and temperature from –4 to 13 °C (lower in the beginning) with cold smoking every third day during the first two weeks (Fig. 1). The production of sausages 50S and 100S ended after 50 days and of sausage 50L after 90 days, when the mass loss was generally higher than 40%.

The measurement of pH values was performed in triplicates on days 0, 4, 7, 10, 20 and at the end of the production (*N*(sample)=54) using a pH-meter IQ 150 (IQ Scientific Instruments, Carlsbad, CA, USA) equipped with the spear type electrode BlueLine 21pH (Schott AG, Mainz, Germany) inserted directly into the sausage samples. Water activity was determined in triplicates (*N*=9) at the end of the production using a portable analyzer HygroPalm HP23-AW-A equipped with a HC2-AW probe (Rotronic AG, Bassersdorf, Switzerland).

### Microbiological analysis and isolation of LAB

Samples for microbiological analysis were taken aseptically in triplicates on days 0, 4, 7, 10, 20 and at the end of the production (*N*=54). Collected sausage samples (25 g) were transferred to sterile plastic pouches and homogenized for 60 s with 90 mL of sterile peptone water (0.1%) using a Stomacher 400 laboratory paddle blender (Seward Ltd., Worthing, UK). Sterile peptone water was also used for the preparation of dilution series, and appropriate dilutions were inoculated in duplicates on agar plates. Total aerobic count was determined on plate count agar (Merck, Darmstadt, Germany) and coagulase-negative staphylococci (CNS) were enumerated on mannitol salt agar (Oxoid, Hampshire, UK) after incubation at 37 °C for 48 h. The Staphaurex® Rapid latex agglutination (Oxoid, Vienna, Austria) served for the confirmation of coagulase-positive staphylococci. *Enterobacteriaceae* were determined on violet red bile glucose agar (Merck) after incubation at 37 °C for 24 h according to ISO 21528-2:2017 (*9*). Chromocult® Coliform ES agar (Merck) was used for the differentiation and enumeration of *Escherichia coli* and coliforms at 37 °C for 24 h according to ISO 4832:2006 (*10*). For the detection of *Listeria monocytogenes,* ISO 11290-1:2017 (*11*), and for *Salmonella* spp. ISO 6579-1:2017 (*12*) standards were applied.

For the isolation, enumeration and collection of LAB isolates, three different media were used: De Man, Rogosa, Sharpe agar (MRS; Sigma-Aldrich Chemie GmbH, Merck, Buchs, Switzerland) supplemented with cycloheximide (0.1 g/L; Sigma-Aldrich Chemie GmbH, Merck) for lactobacilli, Mayeux, Sandine, Elliker agar (MSE; Biolife, Milan, Italy) for *Leuconostoc* species and kanamycin aesculine azide agar (KAA; Biolife, Milan, Italy) for enterococci. MRS and MSE media were incubated under anaerobic conditions at 30 °C for 72 h, whereas KAA was incubated at 37 °C for 48 h. After enumerating MRS, MSE and KAA plates, five to ten colonies were randomly selected, streaked on the corresponding medium and incubated until pure cultures were obtained. Cultures were screened by Gram-staining and stored with 20% (by volume) glycerol at –80 ˚C.

### Fingerprinting and molecular identification of LAB

The template DNA used for all PCR reactions was extracted using the Wizard Genomic DNA Purification Kit (Promega, Madison, WI, USA). All presumptive LAB isolates were genotyped by rep-PCR analysis with the (GTG)_5_ primer (*13*) following the protocol of Domig *et al.* (*14*). The obtained rep-PCR patterns were analyzed using the BioNumerics v. 7.5 software (*15*). The Dice coefficient was used for calculating the genetic similarity of the isolates and the unweighted pair group method with arithmetic average (UPGMA) was used for clustering. A tolerance level of 1.0% and optimization of 0.5% were chosen for creating all dendrograms. Based on cluster analysis, representatives of each cluster were selected and identified by 16S rRNA sequencing (Eurofins MWG Operon, Ebersberg, Germany) by applying the primer set and PCR protocol of Di Cello *et al*. (*16*). Species- or genus-specific PCR was used to confirm the affiliation of all other cluster members at species or, if not possible, at genus level. *Lactobacillus sakei* and *Lactobacillus curvatus* were identified by using primer sets and PCR protocols as described by Berthier and Ehrlich (*17*). *Leuconostoc mesenteroides* and the genera *Weissella* and *Lactobacillus* were identified according to Lee *et al*. (*18*), Schillinger *et al*. (*19*) and Dubernet *et al*. (*20*), respectively. *E. faecalis*, *E. faecium, E. casseliflavus* and *E. gallinarum* were identified by following the PCR protocol of Jackson *et al*. (*21*). All PCR reactions were carried out in a ProFlex PCR system (Applied Biosystems, Foster City, USA). The sequences determined in this study have been submitted to GenBank under accession numbers MG493239-MG493249.

### Sensory analysis

Sensory analysis of ripened dry fermented sausages was performed at the end of the production using a quantitative descriptive analysis method for 21 different attributes. Sausages were tested by 12 panellists (5 females and 7 males, aged 31–54, with more than 5 years of experience in sensory analyses) who were further trained according to ISO 8586:2012 (*22*). Samples were cut into 3-mm thick slices, labelled with random, three-digit codes and served at room temperature on white ceramic dishes. The attributes were scored using a 10-point structured scale, where 0 meant absence of the attribute and 9 meant high intensity of the attribute. Sensory analysis was performed in two sessions following complete block design for balancing the effect of the order of presentation and the carry-over effect. Panellists were instructed to take unsalted bread and tap water before the first sample as well as after each sample for mouth rinsing. Sausages of each type (50S, 100S and 50L) were analyzed in triplicate and the average score for each sample was used for further statistical analysis.

### Statistical analysis

For parametric analysis of pH values and microbial count, the data obtained were analyzed by SAS software (*23*) using generalized linear model (GLM) procedure with least significant difference (LSD) test. NPAR1WAY procedure was used for non-parametric analysis of sensory data with Kruskal-Wallis test and Dwas, Steel, Critchlow-Flinger (DSCF) method at level p=0.05. Results of the parametric and non-parametric analyses are presented as mean value±standard deviation. Principal component analysis (PCA) on correlation matrices with the Varimax rotation was used to find the relationships among sausages of different composition and diameter and the parameters related to sensory attributes and LAB genotypes (XLSTAT-Pro v. 7.5.2 (*24*)). Based on the PCA results, ten strains from the cluster identified as *L. mesenteroides* LM_4 were used for further safety and technological analyses.

### Safety and technological properties of L. mesenteroides strains selected from LM_4 cluster

The haemolytic capacity of ten *L. mesenteroides* strains selected from LM_4 cluster was analyzed on Columbia blood agar (bioMérieux, Craponne, France) and *Bacillus cereus* DSM 6791 served as a positive control. The susceptibility to clinically relevant antibiotics including (in µg): ampicillin 2, clindamycin 2, gentamicin 10, kanamycin 30, tetracycline 5, erythromycin 2 and chloramphenicol 10 was performed by the standardized agar disc diffusion method using BBL™ Sensi-Disc™ antimicrobial susceptibility test discs (Becton, Dickinson and Company, Rungis, France). All strains were inoculated on plates at a concentration equal to the 0.5 McFarland standard. The haemolytic capacity and the susceptibility to antibiotics were evaluated under anaerobic conditions after incubation at 30 °C for 72 h. A PCR assay was carried out to detect the genes encoding for the production of histamine (*hdc*), putrescine (*odc*), tyramine (*tdc*) and cadaverine (*ldc*) as described previously (*25*). The acidifying activity was measured after 24 h and 7 days of incubation at 30 °C by inoculating the strains in lyophilized pork meat media (*26*). The initial pH of the media was pH=5.8. The combined pH electrodes (InPro® 3030; Metter Toledo, Greifensee, Switzerland) were disinfected after each use with 3% HCl. Lipolytic activity was screened on tributyrin agar (Oxoid, Hampshire, UK) by both the disc diffusion and spot method in agar. Bacteria were inoculated (10 μL) on sterile cellulose discs (Biorad, Philadelphia, PA, USA) previously placed on the agar at a cell count corresponding to the 0.5 McFarland standard. At the same time, for the spot method 2 μL of the prepared cell suspension were spotted directly into the agar. Plates were then incubated for 3 days at 30 °C. The radius of zones of clearance formed around the spotting point (spot method) and around the discs (disc diffusion method) was measured and the results were expressed in mm. Proteolytic activity was tested like lipolytic activity, except that instead of tributyrin agar, brain heart infusion agar supplemented with skimmed milk (1.5%) was used. The proteolytic activity of all strains was additionally screened by the well diffusion method using the sarcoplasmic system (*27*). Sarcoplasmic proteins were extracted from lean pork meat as previously described (*28*). Antimicrobial activity of the selected strains was tested against seven indicator bacteria, *Salmonella enterica* (DSM 14221), *Listeria innocua* (ATCC 33090), *Escherichia coli* (ATCC 25922), *Staphylococcus aureus* (DSM 20231), *Brochothrix thermosphacta* (LMG 17208), *Weissella viridescens* (DSM 20410) and *Bacillus cereus* (DSM 6791) using a modified agar streak-spot technique (*14*).

## RESULTS AND DISCUSSION

### Microbiological evolution and pH

On day zero a pH=5.54 was measured in sausages made from a mixture of domestic pig and wild boar meat (50S and 50L) (Table 1), whereas a significantly higher pH value (pH=5.96; p<0.05) was determined in the sausages produced completely from wild boar meat (100S). This is in accordance with differences in the pH values of wild boar and domestic pig meat. It was established that wild boars have higher resistance to short-term stress and have higher number of slower oxidative and lower number of glycolytic fibres resulting in higher pH values (*29*). The pH value of sausage 100S significantly decreased (p<0.05) until day 4. There were no significant pH changes in any sausage type after day 4. Towards the end of the production, there was just a slight pH increase without causing any significant difference in the pH values of the ready-to-eat sausages. The lowest final pH was measured in 50S (pH=5.53), followed by that of 50L (pH=5.57) and 100S (pH=5.64). The water activity values measured at the end of the production were comparable among all three sausage types and ranged from 0.795±0.020 to 0.812±0.020 (data not shown). These results are in agreement with those of Paulsen *et al*. (*30*) and Soriano *et al*. (*7*) and are typical for low-acid sausages ripened for a long time. Regarding the microbiological analysis, similar results were obtained in all three sausage types. Hence, no *Listeria monocytogenes*, *Staphylococcus aureus* or *Salmonella* spp. were detectable at any stage. Also, the number of *E. coli* was below the detection limit (<1.00 log CFU/g) in all sausage types on day zero (0). However, other coliforms and *Enterobacteriaceae* were found at the beginning of the production. Interestingly, the number of these indicator microorganisms was much higher in sausages produced from a mixture of domestic and wild boar meat (4.38 and 4.41 log CFU/g respectively) than in sausages produced only from wild boar meat (3.00 and 2.92 log CFU/g, respectively) (data not shown). The number of these undesirable microorganisms decreased during manufacturing and their count was below the detection limit (<1.00 log CFU/g) in all ripened sausages, indicating the microbiological safety of the ready-to-eat products.

**Table 1:** Number of coagulase-negative staphylococci (CNS), lactic acid bacteria and total mesophilic aerobes (TAC), and pH values determined in wild boar meat sausages during production

*t*/day	Sausage type	*N*/(log CFU/g)					pH
MSA (CNS)	KAA (*Enterococcus* sp.)	MRS (*Lactobacillus* sp.)	MSE (*Leuconostoc* sp.)	PCA (TAC)
0	50S	3.41±0.07	(<1.00±0.00)^b^	(<1.00±0.00)^b^	3.59±0.05	(5.19±0.23)^b^	(5.54±0.04)^b^
	50L	3.46±0.02	(<1.00±0.00)^b^	(<1.00±0.00)^b^	3.59±0.05	(5.19±0.23)^b^	(5.54±0.04)^b^
	100S	3.36±0.04	(3.83±0.07)^a^	(4.81±0.05)^a^	3.65±0.04	(5.95±0.07)^a^	(5.96±0.06)^a^
4	50S	(3.49±0.08)^b^	(3.52±0.07)^a^	(<1.00±0.00)^b^	(5.22±0.17)^a^	(5.22±0.11)^b^	5.56±0.02
	50L	(3.39±0.02)^b^	(3.27±0.02)^b^	(<1.00±0.00)^b^	(4.16±0.00)^b^	(5.55±0.09)^a^	5.46±0.04
	100S	(3.79±0.06)^a^	(3.53±0.01)^a^	(4.79±0.00)^a^	(3.68±0.01)^c^	(5.66±0.07)^a^	5.58±0.01
7	50S	3.56±0.12	(3.04±0.00)^b^	(2.90±0.04)^b^	(3.68±0.04)^b^	(4.98±0.02)^b^	5.53±0.01
	50L	3.58±0.02	(3.37±0.09)^a^	(<1.00±0.00)^c^	(3.57±0.08)^b^	(4.45±0.07)^c^	5.55±0.00
	100S	3.55±0.06	(3.38±0.04)^a^	(4.77±0.01)^a^	(4.23±0.08)^a^	(5.86±0.12)^a^	5.56±0.01
10	50S	(3.49±0.08)^b^	3.17±0.04	(3.18±0.02)^b^	(3.31±0.04)^b^	(4.97±0.00)^b^	5.56±0.01
	50L	(3.44±0.05)^b^	3.04±0.00	(5.00±0.02)^a^	(3.70±0.07)^a^	(5.72±0.05)^a^	5.53±0.02
	100S	(3.67±0.12)^a^	3.15±0.00	(4.98±0.11)^a^	(3.19±0.06)^b^	(5.62±0.09)^a^	5.57±0.01
20	50S	3.32±0.12	(<1.00±0.00)^c^	(3.85±0.07)^b^	(3.37±0.05)^c^	(4.64±0.08)^c^	5.51±0.00
	50L	3.30±0.08	(3.05±0.03)^b^	(3.60±0.03)^b^	(3.68±0.04)^b^	(5.97±0.09)^b^	5.55±0.00
	100S	3.34±0.02	(3.49±0.05)^a^	(6.20±0.06)^a^	(5.72±0.04)^a^	(6.72±0.02)^a^	5.56±0.00
50 or 90	50S	(<1.00±0.00)^a^	<1.00±0.00	(5.96±0.05)^b^	(3.87±0.03)^b^	(6.07±0.01)^b^	5.53±0.00
	50L	(3.52±0.02)^b^	<1.00±0.00	(7.58±0.01)^a^	(3.19±0.03)^c^	(8.07±0.02)^a^	5.57±0.03
	100S	(3.32±0.05)^b^	<1.00±0.00	(6.00±0.06)^b^	(4.19±0.06)^a^	(6.22±0.06)^b^	5.64±0.04

Values are presented as mean±standard deviation. 50S=50% boar meat in small casing, 50L=50% boar meat in large casing, 100S=100% boar meat in small casing, MSA=mannitol salt agar, KAA=kanamycin aesculine azide agar, MRS=De Man, Rogosa, Sharpe agar, MSE=Mayeux, Sandine, Elliker agar, PCA=plate count agar. Within a column, mean values followed by a different letter are significantly different (p<0.05) using the LSD test

The growth trends of the total number of aerobic mesophilic bacteria, CNS and LAB are shown in Table 1. Comparable and stable number of CNS in all three types of sausages was noticed throughout the manufacture. The number of CNS was between 3.36 and 3.46 CFU/g on day 0 and stayed in this range until the end of ripening, except for the ripened sausages of smaller diameter, in which they were not detected. The number of LAB grown on MRS medium slowly increased with ripening. Although they were only detected in sausage 100S on day zero, the number of LAB was comparable in the fully ripened sausages of smaller diameter (5.96 and 6.00 log CFU/g in 50S and 100S, respectively), while they were detected in a much higher number (7.58 log CFU/g; p<0.05) in sausage 50L. The total number of bacteria showed similar trends as LAB grown on MRS agar. On day zero, higher bacterial count (p<0.05) was noticed in 100S sausage (5.95 log CFU/g). At the end of ripening, significantly lower number (p<0.05) was detected in both sausages with a smaller diameter (6.22 and 6.07 log CFU/g in 100S and 50S, respectively) than in that with a larger diameter and a prolonged ripening period (8.07 log CFU/g in 50L). The number of LAB cultured on MSE medium did not differ statistically on day zero but was significantly different (p<0.05) in all end products (3.87, 3.19 and 4.19 log CFU/g in 50S, 50L and 100S sausages, respectively). Enterococci were not detected at the end of the production in any type of ripened sausages. The low growth rate of LAB on MRS and MSE media in all three types of sausages was consistent with the obtained pH=5.53 to 5.64. Contrary to other studies (*5*, *6*), the number of LAB detected in our sausages was very low at the initial stage and remained at lower counts during fermentation and ripening. According to Bover-Cid *et al*. (*31*) fermentation is limited in meat products processed at low temperatures and the pH does not drop more than 0.2–0.4 units. As stated by Spaziani *et al*. (*4*), typical temperatures in the production of dry sausages without starter cultures are below 15 °C. Temperatures in this study were even below 5 °C during the production (Fig. 1) because the process was not designed as controlled but fully artisanal following traditional practices. Although that could reduce the growth of spoilage or pathogenic bacteria, giving positive effect, the growth of beneficial microbiota could also be impaired.

### The effect of sausage diameter and meat batter composition on sensory traits

The effect of sausage diameter and meat batter composition on sensory attributes is shown in Table 2. The sausage filled into a larger diameter casing (50L) had significantly more intense smoke and ripened aroma and was more soluble than the smaller diameter sausage 50S. All acceptability attributes were better rated in sausage 50L than in sausage 50S, with odour and taste acceptability receiving significantly higher scores. Therefore, it could be concluded that the effect of the casing diameter was more pronounced in acceptability traits, but it was not strongly expressed because only five of 21 sensory attributes were significantly different. It has been shown previously that the diameter or product size is an essential parameter that can influence biochemical processes affecting the profile of volatile compounds during ripening by modulating the activities of the endogenous enzymes in meat and the metabolism of the sausage microbiota (*3*). Demeyer (*32*) proposed that this influence is mainly because of changes in physicochemical conditions (*e.g.* kinetics of water loss, extent of acidulation and oxygen availability), which in turn could affect bacterial growth. Therefore, it is assumed that the prolonged ripening time had a major influence on the sensorial properties, resulting in better sensory scores of sausages with larger diameter. This is based on a general conclusion observed by many authors that a higher degree of degradation of proteins, fat and carbohydrates occurs over a prolonged period, which normally affects sensory traits and leads to better acceptance (*4*, *33*, *34*).

**Table 2:** Effects of sausage diameter and meat batter composition on sensory traits of ripened sausages

Trait	Sausage type			Effect of sausage diameter	Effect of meat batter composition
50S	50L	100S
Slice redness	8.22±0.28	7.89±0.11	8.11±0.26		
Colour uniformity	7.22±0.15	7.67±0.24	7.44±0.24		
Fat tissue amount	(4.56±0.45)^b^	(5.56±0.18)^ab^	(6.67±0.37)^a^		**
Slice coherence	8.44±0.24	8.33±0.17	8.00±0.29		
Tenderness	6.78±0.40	7.22±0.15	7.67±0.17		
Solubility	(6.33±0.29)^b^	(7.33±0.17)^a^	(7.78±0.22)^a^	*	**
Saltiness	5.22±0.52	4.56±0.41	4.00±0.33		
Sweetness	1.67±0.44	2.89±0.39	2.78±0.28		
Acidity	1.56±0.24	1.44±0.29	1.11±0.31		
Bitterness	1.56±0.34	0.78±0.28	0.78±0.28		
Smoke aroma	(1.33±0.24)^b^	(2.33±0.24)^a^	(2.22±0.28)^ab^	*	
Aromatic plant aroma	3.33±0.58	1.56±0.47	2.33±0.47		
Spicy aroma	(6.89±0.35)^a^	(5.89±0.51)^ab^	(4.22±0.43)^b^		**
Ripened aroma	(6.11±0.35)^b^	(7.56±0.18)^a^	(6.78±0.22)^b^	**	
Off-flavours	1.44±0.38	0.44±0.18	0.56±0.24		
Slice acceptability	7.11±0.20	7.89 ±0.26	7.11±0.26		
Odour acceptability	(6.44±0.41)^b^	(8.22±0.28)^a^	(6.89±0.11)^b^	**	
Texture acceptability	7.44±0.38)	8.22±0.28	7.33±0.17		
Taste acceptability	(6.22±0.52)^b^	(7.89±0.31)^a^	(6.78±0.28)^b^	*	
Aftertaste	7.22±0.32	8.00±0.24	6.78±0.15		
Overall acceptability	6.78±0.28	7.78±0.28	6.67±0.24		

Values are presented as mean±standard deviation. 50S=50% boar meat in small casing 50L=50% boar meat in large casing, 100S=100% boar meat in small casing. Within a row, mean values followed by a different letter are significantly different (*p<0.05, **p<0.01) according to the Kruskal-Wallis test

Statistical analysis of meat batter composition revealed that the amount of fat tissue and solubility were rated significantly higher in sausages 100S produced only from wild boar meat, while the spicy aroma was evaluated higher in sausage 50S made from wild boar and domestic pig meat (Table 2). Acceptability traits were not significantly different between sausages 50S and 100S. Although odour and taste acceptability of sausage 100S were rated higher, sausage 50S received higher evaluation of texture, aftertaste and overall acceptability. Similar results were obtained in the study of Paulsen *et al.* (*30*), where mould ripened sausages produced from a mixture of wild boar meat and domestic pig fat performed significantly better than sausages made from wild boar meat only. The same authors found that sausages with fat tissue from wild boar had two to three times higher thiobarbituric acid reactive substances (TBARSs) and peroxide values (POVs) which imply higher oxidation rate and could lead to worsening of some aroma sensory traits and especially acceptability. Higher TBARS and POVs in sausages manufactured with fat tissue from wild boar could be the consequence of a higher concentration of unsaturated fatty acids, as reported by Sales and Kotrba (*29*).

### Relationships between the sausage types, their sensory traits and predominant LAB genotypes

In this study, a total of 409 LAB isolates was collected. In all sausage types, similar composition of LAB species was noticed. The most frequently isolated species was *Leuconostoc mesenteroides* (44.99%), followed by *Enterococcus casseliflavus* (30.81%) (data not shown). These two species represented the clear majority of isolates obtained from 50S (74.83%) and 50L sausages (90.40%). In the sausage produced only from wild boar meat (100S), a slightly higher diversity was noted with 63.16% of isolates belonging to the predominant species *L. mesenteroides* and *E. casseliflavus* followed by *Lactococcus garvieae* (9.77%) and *Weissella viridescens* (8.27%). The observed differences between different sausage types may be mainly due to the microbiota of the raw materials, as already described by Talon *et al.* (*35*), and less the result of cross-contamination between the environment and the meat. Usually, *Lactobacillus* spp. are described as the dominant LAB in fermented sausages (*36*). The high prevalence of *L. mesenteroides* is considered controversial from a technological point of view (*1*); however, it is well documented in sausages containing up to 2.5% of salt (*8*, *37*, *38*), as confirmed in our study.

**Fig. 2:**
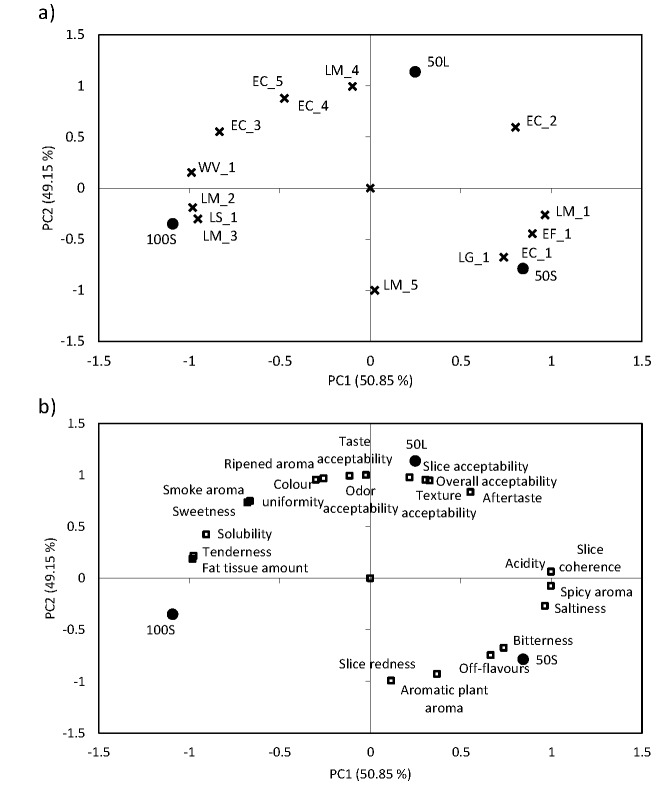
Sausage scores on the plot of the first two principal components (PC1 *vs* PC2): a) bacterial cluster loadings, b) sensory trait loadings. 50S=50% wild boar meat in small casing, 50L=50% wild boar meat in large casing, 100S=100% wild boar meat in small casing, LM=*Leuconostoc mesenteroides*, EC=*Enterococcus casseliflavus*, LG=*Lactococcus garvieae*, WV=*Weissella viridescens*, LS=*Lactobacillus sakei*, EF=*Enterococcus faecalis*

Although no particular differences were found among the tested sausages regarding species distribution, cluster analysis was performed to obtain more information at strain (genotype) level. Hence, all collected LAB isolates were clustered based on their rep-PCR patterns, and discrimination was only considered if the genotypes were less than 90% similar. To explore the possible relationships between the sausage types, their sensory traits and bacterial genotypes, only clusters containing five or more strains were selected for further analysis. Therefore, respectively one *L. garivieae* (LG_1), *W. viridescens* (WV_1), *E. faecalis* (EF_1) and *L. sakei* (LS_1) cluster as well as five *L. mesenteroides* (LM_1-LM_5) and five *E. casseliflavus* (EC_1- EC_5) clusters were used for the principal component analysis (PCA) (Table 3). In total, PCA was carried out on the correlation matrix based on the 21 sensory variables and 14 bacterial clusters. The first two principal components (PCs) accounted for 100% of variance (PC1=50.85%, PC2=49.15%; Fig. 2). The measurements and the PCs were interpreted according to the correlations between each parameter and each PC. The most important variables for PC1 were four sensory traits (slice coherence, acidity, spicy aroma and saltiness) and one bacterial cluster LM_1 that correlated positively, while three sensory traits (fat tissue amount, solubility and tenderness) and four bacterial clusters (WV_1, LM_2, LS_1 and LM_3) correlated negatively. PC2 was mostly defined by seven sensory traits (taste, odour, slice, texture and overall acceptability, and ripened meat aroma and colour uniformity) and one bacterial cluster LM_4 that correlated positively, as well as two sensory traits (slice redness and aromatic plant aroma) and one bacterial cluster LM_5, which correlated negatively. It was found that 16 of 21 sensory variables and seven of 14 bacterial clusters were important for the characterization of the PCs.

**Table 3:** Number of genotypes in a cluster and in sausage types

Species identification	Cluster	*N*(genotype)			
Cluster	50S	50L	100S
*Leuconostoc mesenteroides*	LM_1	112	63	36	13
	LM_2	9	0	1	8
	LM_3	11	0	0	11
	LM_4	29	0	22	7
	LM_5	13	6	2	5
*Enterococcus casseliflavus*	EC_1	6	6	0	0
	EC_2	5	2	3	0
	EC_3	11	0	5	6
	EC_4	5	0	3	2
	EC_5	6	0	5	1
*Lactococcus garvieae*	LG_1	12	12	0	0
*Weissella viridescens*	WV_1	19	2	6	11
*Enterococcus faecalis*	EF_1	9	7	2	0
*Lactobacillus sakei*	LS_1	8	0	0	8

Values are presented as total number. 50S=50% wild boar meat in small casing, 50L=50% wild boar meat in large casing, 100S=100% wild boar meat in small casing

Several bacterial clusters were important for the PC characterization but all *L. mesenteroides* clusters were important for both, positive and negative sides of the PC1 and the PC2, suggesting a high variability and ambiguous role of this species in the production of sausages. The versatile involvement of the same LAB species in the sensory characterization of sausages can be explained by the fact that many metabolic traits that contribute to aroma development are strain-specific (*2*), which is especially true for *L. mesenteroides* in this study. As a representative of the LAB, *L. mesenteroides* can promote fermentation and certain genotypes may also act as bio-preservative due to antilisterial activity, but they may also be associated with food spoilage and cause off-odours, slime formation and bloating of sausages due to their heterofermentative metabolism (*1*). Therefore, their role still needs to be resolved to determine if they are involved in product spoilage or have beneficial effect on sausage quality due to the rapid acidification, production of bacteriocins, acetaldehyde, diacetyl and ethanol (*37*, *39*). Two other species were also found to be relevant for PC characterization, namely *W. viridescens* (cluster WV_1) and *L. sakei* (cluster LS_1), both on the negative side of PC1.

As seen in Fig. 2, sausage 100S was clearly separated from sausage 50S along PC1, while sausage 50L was separated from sausages 50S and 100S along PC2. In addition to the differences in their sensory properties (Table 2), it was found that the tested sausages could also be distinguished by the present LAB genotypes, although no significant differences in the composition of the LAB species were detected. Isolates of clusters LG_1 and EC_1 were mainly determined in sausage 50S and those of clusters LS_1, LM_2 and LM_3 in sausage 100S. Because sausages 50S and 100S consisted of different meat batter, these results correspond with conclusions drawn by Talon *et al.* (*35*), who found no evidence of cross-contamination between the environment and the meat, assuming that the microbial count of the batter was mainly due to the microbiota of raw materials. Sausage 50L was close to PC2, where LM_4 cluster had high loading. Furthermore, it was established that sausage 50L was closely related to sensory traits, especially those regarding acceptability (slice, texture and overall acceptability). This could lead to the conclusion that different LAB genotypes were related to the particular sausage types although the dominant bacteria (*L. mesenteroides*) had an influence on the sensory properties of all tested sausages. Furthermore, the longer ripening time can favour the growth of slow growing LAB or certain LAB genotype, and 50L sausages seem to be closely linked to one *L. mesenteroides* genotype (LM_4) and overall acceptability traits.

Because of its correlation to positive sensory properties, overall safety and technological properties of LM_4 genotype were tested (Table 4). Within this, ten strains of the same LM_4 cluster (containing 29 strains) were tested to assess possible phenotypic variability of the same genotype. The results of the safety analysis and the metabolic profiles of the selected LM_4 strains are presented in Table 4. All strains were susceptible to all tested antibiotics, possessed no genes for biogenic amines and showed no haemolytic activity (data not shown). Technologically important traits and bioprotective roles were also considered, primarily acidification and antagonistic activity, but also lipolytic and proteolytic activity, as they are the most desirable technological features for functional starter cultures (*40*). All strains exhibited some degree of proteolytic activity on skimmed milk but no lipolytic activity and no proteolytic activity on sarcoplasmic proteins were found. However, they showed noticeable acidification ability (p<0.05), lowering the pH from the initial pH=5.41 to an average value of pH=4.01 and 3.74 after 24 h and 7 days of incubation, respectively (Table 4). All tested strains had antagonistic activity against *Staphylococcus aureus* ssp. *aureus* and 50% of them against *Brochothrix thermosphacta*. Despite the absence of some important technological properties like lipolytic and proteolytic activity on sarcoplasmic proteins, high correlation with overall acceptability traits, high acidification potential and antagonistic activity make *L. mesenteroides* LM_4 genotype interesting for further application as starter or adjunct culture.

**Table 4:** Proteolysis, acidification and antagonistic traits of ten *Leuconostoc mesenteroides* (LM_4) strains belonging to the same genotype (cluster)

Strain code	Proteolysis (*d*/mm)		Acidification (pH)		Indicator bacteria	
Disc diffusion method	Spot method	*t*=24 h	*t*=7 day	*Staphylococcus aureus* ssp. *aureus*	*Brochothrix* *termospachta*
LM_4 G1_1	14.50±0.71	11.00±0.00	3.99±0.01	3.74±0.02	+	–
LM_4 G1_8	15.00±1.41	10.00±0.00	3.98±0.04	3.74±0.02	+	–
LM_4G1_10	14.00±0.00	11.00±0.00	4.09±0.02	3.75±0.02	+	+
LM_4 G1_11	14.00±0.00	11.00±0.00	4.06±0.04	3.75±0.02	+	–
LM_4 G1_3	15.00±0.00	9.00±0.00	3.99±0.02	3.73±0.02	+	+
LM_4 G1_7	14.50±0.71	11.00±0.00	4.01±0.00	3.76±0.01	+	–
LM_4 G1_13	15.00±1.41	10.00±0.00	4.02±0.02	3.78±0.01	+	+
LM_4 G1_15	14.00±0.00	11.00±0.00	4.06±0.00	3.77±0.02	+	+
LM_4 G1_5	14.00±0.00	11.00±0.00	3.98±0.02	3.71±0.01	+	–
LM_4 G1_9	15.00±0.00	9.00±0.00	3.97±0.01	3.72±0.04	+	+

Values are presented as mean±standard deviation. Results of antagonistic activity are expressed as the difference in the measured diameters, where –=no inhibition (the difference in their diameters is <1 mm), and +=inhibition (grown colonies are 1–2 mm smaller than the growth control)

## CONCLUSIONS

The results of this study showed a partial effect of casing diameter and meat batter composition on the load of the microbiota, count of different LAB members and pH values. Effect of casing diameter and meat batter composition on sensory traits was evident regardless of the same additives and manufacture procedure. Principal component analysis (PCA) was proven to be a helpful tool for explaining the data which are mutually but not obviously related. It was found that *Leuconostoc mesenteroides* genotypes were important for the characterization of both, positive and negative sides of PCs, suggesting an ambiguous role of this species in sausage production. PCA showed that sensory traits and bacterial cluster analysis could be used for differentiating sausages with various diameters and meat batter compositions. Even more, the two sausages with a smaller diameter could be distinguished by specific bacterial clusters. As such, this research demonstrates how PCA analysis can be used to relate product sensory properties to particular microbial genotype. Accordingly, specific microorganisms could be selected for potential use as starter or adjunct culture in standardization of sausages production.
